# Factors Affecting Measurement of Salivary Cortisol and Secretory Immunoglobulin A in Field Studies of Athletes

**DOI:** 10.3389/fendo.2017.00168

**Published:** 2017-07-24

**Authors:** Barry Thomas Pritchard, Warren Stanton, Roger Lord, Peter Petocz, Gert-Jan Pepping

**Affiliations:** ^1^School of Physiotherapy, Australian Catholic University, Brisbane, QLD, Australia; ^2^School of Science, Australian Catholic University, Brisbane, QLD, Australia; ^3^Department of Statistics, Macquarie University, Sydney, NSW, Australia; ^4^School of Exercise Science, Australian Catholic University, Brisbane, QLD, Australia

**Keywords:** stress, cortisol, sIgA, salivary immunoglobulin A, biomarkers, salivary cortisol, stress hormone, HPA axis

## Abstract

**Aims:**

Biological and lifestyle factors, such as daily rhythm, caffeine ingestion, recent infection, and antibiotic intake, have been shown to influence measurements of salivary cortisol (SC) and secretory immunoglobulin A (sIgA). Current methodology in unsynchronized, field-based biomarker studies does not take these effects into account. Moreover, very little is known about the combined effects of biological and lifestyle factors on SC and sIgA. This study supports development of a protocol for measuring biomarkers from saliva collected in field studies by examining the individual and combined effects of these factors on SC and sIgA.

**Method:**

At three time points (start of the pre-season; start of playing season; and end of playing season), saliva samples were collected from the entire squad of 45 male players of an elite Australian Football club (mean age 22.8 ± 3.5 years). At each time, point daily rhythm and lifestyle factors were determined *via* a questionnaire, and concentrations of both SC and sIgA *via* an enzyme linked immuno-sorbent (ELISA) assay of saliva samples. In addition, player times to produce 0.5 mL of saliva were recorded.

**Results:**

Analysis of covariance of the data across the three time points showed that daily rhythm had a more consistent effect than the lifestyle factors of caffeine ingestion, recent infection, and antibiotic intake on SC, but not on sIgA. Data for sIgA and SC concentrations were then adjusted for the effects of daily rhythm and lifestyle factors, and correlational analysis of the pooled data was used to examine the relative effects of these two sources of influence on sIgA and SC. With the exception of time to produce saliva, the biological measures of stress were affected by players’ daily rhythms. When daily rhythm was taken into account the group of lifestyle factors did not have an additional effect.

**Discussion:**

It is recommended that future studies measuring SC and sIgA make additional adjustments for the daily rhythm, in particular time since first sight of daylight, as small measurement errors of biomarkers can confound discrimination among study participants.

## Introduction

Stress manifests by diverse etiologies with many undesirable health outcomes, including effects upon performance in sport, as well as other occupational and performance contexts. Major to minor health effects of stress have been reported (1), and in a sports environment, levels of stress have been linked to recovery time, burnout due to overtraining, muscle damage repair time, and susceptibility to infection (2–7).

Psychological and physiological stressors are important instigators of hypothalamus–pituitary–adrenal axis activation, which have downstream influence on parameters such as immunoglobulin levels and salivary cortisol (SC) concentrations in humans. For this reason, SC, and secretory immunoglobulin A (sIgA) are widely reported as biomarkers of stress (8). Previous studies of stress biomarkers have not adequately adjusted SC and sIgA for biological effects such as daily rhythm and lifestyle factors including caffeine ingestion, recent infection, and antibiotic intake (8,  9). For example, adjustment for daily rhythm is performed by collecting samples at similar times, but no studies could be identified that took into account daily rythym variation in participants’ first seeing daylight after waking. To assist in the development of a protocol for measuring biomarkers from saliva collected in field studies, the aim of the current study was to investigate the combined effects of daily rhythm and lifestyle factors, specifically: caffeine ingestion, recent infection, and antibiotic intake, on SC and sIgA collected from a population of high performance athletes.

Cortisol and sIgA concentrations can be obtained *via* blood sampling, though the inherent risks associated with venipuncture are numerous (10). Previous research has shown that SC is a more robust measure of stress than serum cortisol because SC is a better measure of adrenal cortical function (11, 12). Saliva sample collection is non-invasive, more time efficient, and safer in field studies than blood collection. For this reason, saliva sample collection has become more widely used for measurement of SC and sIgA in the context of stress in research fields such as sports exercise physiology and biopsychology.

In terms of biological factors that affect measures of stress, daily rhythms have been reported to affect SC and sIgA concentrations and associated salivary flow rate (13). It is well understood that the first sight of daylight after waking initiates the circadian response although there is surprisingly little research available on daily rhythm variation within normal salivary flow rate over a 24-h period.

A number of lifestyle factors have been shown to confound measurement of SC and sIgA (8). In the current study of a homogenous group of elite athletes, we will focus on the most prevalent confounding influences of caffeine ingestion, recent infection, use of antibiotics, and recent surgery (14). Caffeine, a commonly ingested psychoactive agent found in coffee, tea, and energy drinks has been shown to elevate concentrations of SC (15). Ingestion of antibiotics and/or recent infection has been associated with an increased SC and sIgA production (16). An increase in sIgA occurs after antigenic stimulation *via* the humoral immunity response, which varies within individuals and type of antigen exposure. For example, influenza sIgA antibodies usually peak two weeks after stimulation in the majority of the population (17). Recent surgery has been shown to increase SC, likely as a stress response from the body to produce a higher prophylactic concentration of anti-inflammatory agent (14, 18, 19). Surprisingly, in many previous studies, the confounding effects of these lifestyle factors are not taken into account (20, 21).

When working with sports teams, it is often difficult to fit a testing protocol into their strict training regime. Players awake at different times of the morning, and often arrive at the athletic club to train in prearranged staggered groups. Dawes (22) showed that daily rhythms also affect the time taken to produce unstimulated saliva. This implies that measurement of SC, sIgA, and salivary flow rate should be standardized by regression to a common time point to adjust for the time differential since participants first saw daylight (22). A similar argument can be developed with regards to other factors that influence levels of SC and sIgA. It is likely that players have different levels of caffeine ingestion and rates of infection and antibiotic use, indicating a need to assess and adjust for these individual differences.

The development of reliable SC and sIgA quantification research methods for biomarker studies is imperative, to improve of the quality of studies of stress and the information used to assist medical care, coaching style, and player development of athletes. The rationale for this study was that a significant error may be introduced if measures are not adequately corrected for biological and lifestyle factors, in particular daily rhythm. The aim of this study, therefore, was to examine the effects of daily rhythm, caffeine ingestion, recent infection, and antibiotic intake on SC and sIgA concentrations. In particular, the study; (a) examined consistency in the effect of biological and lifestyle factors across three time points, and (b) compared the combined and separate effects of these factors.

## Materials and Methods

### Sample

Participants in the study were a cohort of 46 elite male players from a club in the National Australian Football League, representing the entire training squad of the club. The study was approved by the Human Research Ethics Committee, Australian Catholic University (HREC ACU), and consent obtained from all research participants was both informed and written. The mean (±SD) age, height, and weight of the players was 22.8 ± 3.5 years, 187.9 ± 6.0 cm, and 88.3 ± 6.6 kg, respectively. Players’ ages ranged from 19 to 31 years.

### Procedures

Questionnaires were administered at three time points, the start of pre-season (T1), the start of the playing season (T2), and the end of the playing season (T3). Players provided information about demographic characteristics, recent injury history, time of first seeing daylight, caffeine ingestion before they arrived at the sports club, illness or infection in the past week, antibiotic ingestion in the past week, and surgery in the past month. Saliva samples were collected at each assessment time point.

Players arrived at the club in staggered groups of four. A sampling team of five experienced researchers were trained in the sampling procedure to ensure consistent technique. One member of the team was present at the point of entry to record arrival times and to ensure all players thoroughly rinsed their mouth with water. Players were requested to give a saliva sample 10 min after the rinse as per the immunoassay protocol (Salimetrics, Carlsbad, CA, USA).

Players were seated in a quiet room, where visual and verbal contact with other players was minimized. Research staff informed each player of the process required for passive saliva collection, and answered any participant questions. Unstimulated saliva was collected into a small pre-labeled Eppendorf tube *via* a straw, and the time taken to obtain 0.5 mL of saliva was recorded in seconds.

The saliva samples were immediately stored at approximately 4°C in a polystyrene container with ice, prior to transport and subsequent storage in the laboratory. The sample was de-identified by application of a laboratory number, sealed in a plastic snap lock bag, and chilled to prevent both microbial growth and protein degradation.

Within the testing laboratory, samples were split into two Eppendorf tubes to allow assay re-run if required. Labeled saliva samples were frozen at −80°C within a secure, back-up powered ultra-low freezer within 4 h of collection. No additional preservatives such as sodium azide were added to the samples to exclude possible assay interference. Freezing saliva samples are known to precipitate mucins within the saliva samples so before assaying, samples were thawed, vortexed, and centrifuged at 1,500 g (@3,000 rpm) for 15 min as per ELISA protocol (Salimetrics, Carlsbad, CA, USA). The sIgA and SC ELISA assays were both read using a Biorad iMark microplate reader with a 450 nm filter (23).

### ELISA Assays

To quantify the steroid hormone SC and sIgA proteins, saliva samples were taken and assayed using competitive ELISA kits from Salimetrics (Carlsbad, CA, USA) and used in accordance with the manufacturer’s directions.

#### Adjustment for the Effects of Daily Rhythm on Cortisol and IgA Concentrations

Salivary cortisol and sIgA diurnal fluctuation data [taken from Ref. (24)] were used to adjust cortisol concentrations to a standardized time of first daylight response. First, originally measured participant concentrations of SC were transformed to square root nmol/L, which for the remainder will be referred to as the unadjusted cortisol concentration (SC_unadjusted_). From the time of seeing first daylight for the individual participant and the diurnal cycle data presented by Hucklebridge et al. (24), an individual adjustment concentration value (SC_adjust_) was obtained. To calculate the adjusted SC concentration since awakening (Daily Rhythm Adjusted: SC_DRA_), the individual adjustment concentration was added to the SC_unadjusted_. In short:

(1) $${\text{SC}}_{\text{DRA}}={\text{SC}}_{\text{unadjusted}}+{\text{SC}}_{\text{adjust}}$$

The same process was followed for sIgA. As Hucklebridge et al. (24) described diurnal sIgA response in concentration values of μg/mL, no transformation of units was necessary for sIgA. The adjusted sIgA concentration since awakening (Daily Rhythm Adjusted: sIgA_DRA_) was calculated as the sum of the unadjusted sIgA concentration (sIgA_unadjusted_) and the individual adjustment concentration value (sIgA_adjust_), calculated from the time since awakening for the individual participant and the diurnal cycle data presented by Hucklebridge et al. (24).

(2) $${\text{sIgA}}_{\text{DRA}}={\text{sIgA}}_{\text{unadjusted}}+{\text{sIgA}}_{\text{adjust}}$$

#### Adjustment for the Effects of Lifestyle Factor on Cortisol and IgA Concentrations

Adjustment for the lifestyle factors was achieved by subjecting SC_DRA_ and sIgA_DRA_ to regression analysis, to obtain the means and unstandardized residual variances. The residuals were then added to the respective means to calculate lifestyle adjusted ${\text{SC}}_{\text{DRA}}^{\text{LA}}$ and ${\text{sIgA}}_{\text{DRA}}^{\text{LA}}$ with the effect of daily rhythm and lifestyle factors removed.

### Statistical Analysis

#### Consistency of Factors across Time

Analysis of covariance (ANCOVA) with Type III sums of squares model (SPSS, version 22; www.spss.com) was used to examine the effects of the daily rhythm and lifestyle factors on the biomarker measures of stress at three separate times. The outcome measures were SC_unadjusted_ and sIgA_unadjusted_, and salivary flow rate. The concentrations of sIgA and cortisol were analyzed on a log scale in order to satisfy the distributional assumptions required for the analyses. Age, height, and weight were included as covariates in the analysis. The independent variables of interest were caffeine ingestion on the day of testing, infection in the past week, and use of antibiotics in the past week (all coded no or yes), as well as time since seeing daylight to collection of saliva (minutes), and time to produce 0.5 mL of saliva (seconds).

#### Relative Effects of Daily Rhythm and Lifestyle Factors

The data for the three time points were pooled (*n* = 130) to provide an average effect across time. Correlational analysis was used to assess the effect of daily rhythm and lifestyle factors on the unadjusted and the adjusted measures of SC and sIgA concentrations. Percentage change in the correlation was used to estimate the size of the confounding effect.

## Results

### Consistency across Time

Among the 130 assessments across the three time periods, caffeine consumption before arriving at training occurred on 22.0% of occasions, an infection in the past week occurred on 31.8% of occasions, and use of antibiotics in the past week occurred on 24.2% of occasions. None of the players had recent surgery. There was consistency across time for the rate of caffeine consumption and use of antibiotics, but the rate of infection was higher on the third assessment day (51.2%) compared to other days (21.7 and 23.3% respectively; chi-square = 11.02, *p* = 0.004).

The ANCOVA results shown in Table 1 indicate that the biological factors of “time since seeing daylight” and “time to produce 0.5 mL of saliva” were associated only with SC_unadjusted_. The measure of daily rhythm (time since seeing daylight) was the only factor related to a biomarker at more than one time point. Some lifestyle factors were associated with SC_unadjusted_ and sIgA_unadjusted_ concentrations when players returned after the off-season (Time 1) but not after the pre-season or playing season.

**Table 1:** Factors related to concentrations of cortisol and immunoglobulin A in saliva.^a^^,^^b^

Measure	Time 1 (*n* = 44) start pre-season		Time 2 (*n* = 43) start season		Time 3 (*n* = 43) end season	
			
	*F*	*p*	*F*	*p*	*F*	*p*
	**SC_unadjusted_**					

Caffeine (today)	0.32	0.58	0.01	0.97	0.14	0.71
Infection or illness (last week)	0.36	0.55	0.39	0.54	0.03	0.86
Antibiotics (last week)	6.17	0.02*	0.14	0.71	0.98	0.33
Caffeine × infection	0.89	0.35	0.49	0.49	0.12	0.73
Caffeine × antibiotics	0.45	0.51	0.13	0.73	0.88	0.36
Infection × antibiotics	6.13	0.02*	0.12	0.74	0.52	0.48
Time since seeing daylight (min)	1.55	0.22	5.70	0.02*	4.88	0.04*
Time for saliva^c^ (s)	8.36	0.01*	0.45	0.83	0.02	0.88

	**sIgA_unadjusted_**					

Caffeine (today)	0.01	0.99	0.09	0.77	1.09	0.30
Infection or illness (last week)	0.19	0.67	0.07	0.79	1.09	0.30
Antibiotics (last week)	2.32	0.14	0.41	0.53	0.27	0.61
Caffeine × infection	0.78	0.38	1.59	0.22	0.74	0.40
Caffeine × antibiotics	4.94	0.03*	0.15	0.70	0.24	0.63
Infection × antibiotics	4.62	0.04*	0.48	0.49	0.05	0.82
Time since seeing daylight (min)	0.03	0.87	0.09	0.77	0.16	0.69
Time for saliva^c^ (s)	0.29	0.59	0.77	0.39	0.13	0.72

^a^Adjusted for age, height and weight, *p < 0.05.

^b^Lifestyle factors of caffeine, antibiotics, and infection coded “yes” or “no.”

*^c^Time to produce 0.5 mL of saliva*.

### Relative Effects of Biological and Lifestyle Factors on Measures of Stress

**Figure 1:**
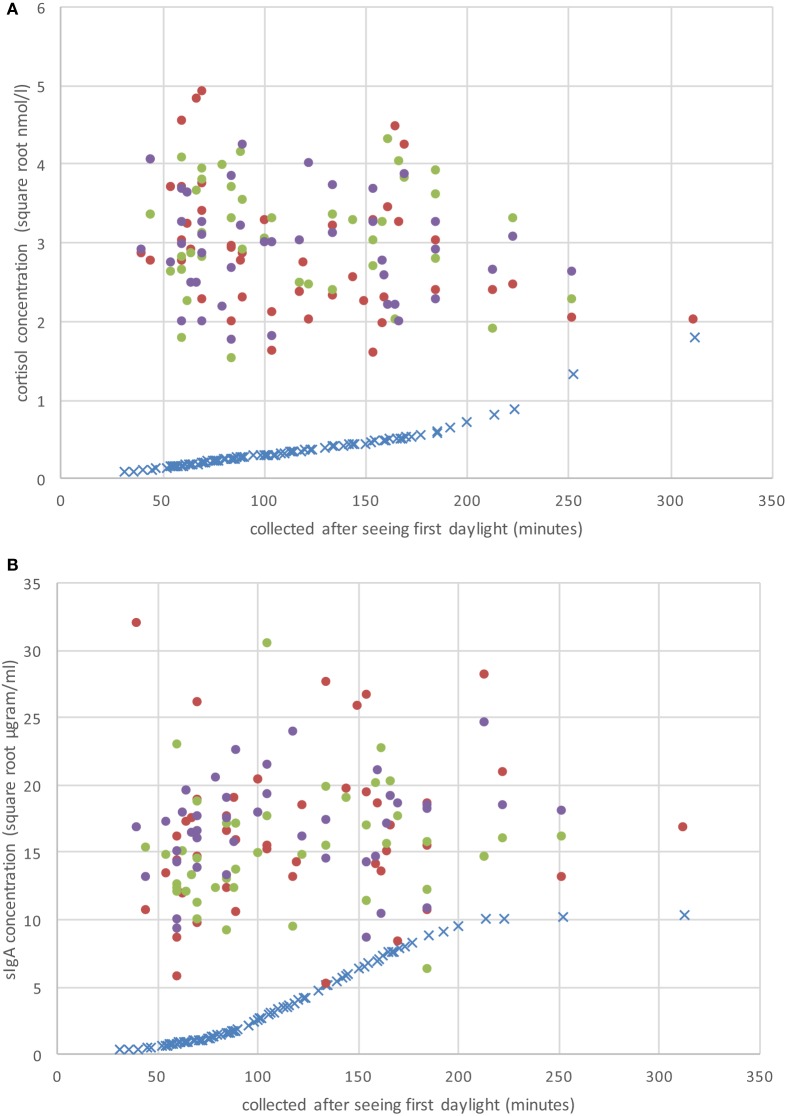
Raw CS **(A)** and secretory immunoglobulin A (sIgA) **(B)** concentrations on the three sample periods (day 1 = solid red dots; day 2 = solid green dots; day 3 = solid purple dots), and the individual adjustment concentration value (blue “×”) based on Hucklebridge et al. (24) as a function of when the sample was collected after seeing first daylight (in minutes).

The Pearson correlation statistics shown in Table 2 for the pooled data indicate the extent of change from adjusting SC and sIgA concentrations for the effects of saliva flow rate, daily rhythm, CAI (caffeine consumption, infection, antibiotic use), and the combination of these factors. The time to produce 0.5 mL of saliva was least affected by the daily rhythm (1%). SC and sIgA concentrations changed by 5% or more due to the daily rhythm (Column 1 of Table 2). The table values in Column 2 of Table 2 indicate that after adjustment for daily rhythm, adjustment for lifestyle factors made little or no further difference to the magnitude of correlation. A descriptive comparison of the original and adjusted values used for analysis is provided in Figure 1 and Table 3.

**Table 2:** Correlations of biomarker measurements of stress (*n* = 130).

Measure (adjustment)	Units	Adjustment	
		Daily rhythm	Daily rhythm + lifestyle factors
Time for saliva^a^^,^^b^	s	0.99	0.99
SC	μg/dL	0.95	0.94
sIgA	μg/mL	0.93	0.93

*Daily rhythm = adjusted for daily rhythm*.

*Lifestyle factors = caffeine (today), antibiotics (last week), infection (last week)*.

*^a^Time = time to produce 0.5 mL of saliva*.

*^b^Time since first sight of daylight used to represent the daily rhythm*.

**Table 3:** Descriptive statistics of biomarker measurement of stress (*n* = 130).

Measure (adjustment)	Units	Mean	SD	Minimum	Maximum
Time	s	151.87	112.07	10.00	480.00
Time (daily rhythm)	s	167.56	112.63	15.45	487.50
Time (daily rhythm + lifestyle)	s	167.56	110.62	27.04	476.83
SC_unadjusted_	μg/dL	0.34	0.16	0.08	0.86
SC_DRA_	√nmol/L	3.30	0.68	1.84	5.10
${\text{SC}}_{\text{DRA}}^{\text{LA}}$	μg/dL	3.30	0.68	1.87	5.08
sIgA_unadjusted_	μg/mL	287.96	180.07	25.99	1,030.68
sIgA_DRA_	√μg/mL	19.40	5.76	6.49	37.99
${\text{sIgA}}_{\text{DRA}}^{\text{LA}}$	μg/mL	19.40	5.75	6.21	37.96

*Time = time to produce 0.5 mL of saliva*.

*Lifestyle = Caffeine (today), antibiotics (last week), infection (last week)*.

## Discussion

Biomarkers are commonly used as measures of physiological reactions to stress, of which SC and sIgA are highly regarded. In the present study, development of a procedure to measure biomarkers of stress in field studies was conducted with a homogenous sample of fulltime sportsmen. We studied Australian Football players in a single team, with similar levels of environmental stressors such as training regimes and competition match play, and similar levels of psychological pressure related to the competitiveness of playing professional football. This enabled us to investigate the effects of daily rhythm and lifestyle factors on SC and sIgA.

### Effects of Lifestyle Factors

A relationship between lifestyle factors and biomarkers of stress was evident when players returned after the off-season, but not during the training and playing seasons. This relationship was represented mostly by interaction effects, for example combined effects of infection and antibiotic use (Table 1). This may have been due to changes in the intensity of the athletes’ training regime (8), or a change in prevalence of the lifestyle factors. In addition, changes in individual IgA antibody responses may be related to the particular type of infection, indicating a need for future studies to investigate the relationship between stress response and type of infection. The absence of an effect of caffeine intake may be due to regular caffeine consumption producing tolerance effects. Research has shown that tolerance to caffeine is related to a reduced cortisol response (15).

In the analysis of the pooled data (Table 2), the biological factor of daily rhythm was taken into account before the lifestyle factors, as it was more consistently associated with levels of biomarker concentrations across time. After adjustment for this biological factor, the lifestyle factors no longer affected the degree of association with SC and sIgA. These findings suggest that field studies may not need to adjust for these lifestyle factors. Confirmation of this could be obtained by a study of the effect of lifestyle factors on biomarker concentrations, when saliva samples are collected at the same time relative to first sight of daylight.

### Effects of Daily Rhythm

Analysis of the biomarkers at three separate time points indicated that daily rhythm, measured as time of saliva collection relative to first sight of daylight, was not related to sIgA concentrations but was significantly associated with cortisol concentrations during the periods over the playing season. A proxy measure of the effect of daily rhythm—time since seeing daylight—was used and has potential validation in the first part of the analysis (results in Table 1) as there are no published biological studies reporting the effect of daily rhythm on production of saliva. In the second part of the analysis (Table 2), correlation analysis of the pooled data indicated that the SC and sIgA were affected by daily rhythm with the exception of time to produce saliva (Column 1 of Table 2).

A measure of time from participants’ first sight of morning daylight is simpler to apply to concentrations of stress biomarkers than transformations based on the non-linear effect of the diurnal cycle reported by Dawes (13) and Hucklebridge et al. (24). Although there is a high correlation between the linear measure of time, used in the first analysis, and the curvilinear measure of daily rhythm used in the second analysis, it should be noted that use of the linear measure is associated with a small measurement error. On the other hand, the reported studies of the relationship between biomarkers and the circadian and diurnal cycle had small sample sizes and may also have contained measurement error. Further large sample and longitudinal studies are required to more accurately map the effect of daily rhythm on SC and sIgA.

The measure of time to produce a set amount of saliva production was found to be related to cortisol concentrations. Concentrations of cortisol are commonly reported to be independent of the rate of saliva production (Salimetrics Assay, Carlsbad, CA, USA). However, a measure of time to produce saliva could be an alternative biological measure of stress, as suggested by the results of Ref. (25). Further evidence of this relationship may provide a simpler, less invasive measure of stress than collection of saliva and should be investigated further as a proxy measure of stress.

### Other Factors

It has been reported that gender affects susceptibility to stress and daily rhythms regarding salivary flow rate, sIgA, and SC release (26). These effects should be considered in future studies of female athletes. In addition, glucocorticosteroid administration by oral, intravenous, intramuscular, and rectal routes may affect assay concentrations of SC or sIgA. Other differences related to the measures of stress are likely to reflect changes in other factors such as training regimes, and warrant further controlled studies to examine the source of this variability.

## Conclusion

Development of a protocol for accurate measurement of SC and sIgA should take into account the effects of daily rhythm. Obtaining research data from sports teams often requires unsynchronized access to a cohort of team members due to strict training regimes. The data from this study showed that adjustment of cortisol concentrations to account for an individual’s daily rhythm is warranted. Staggered sampling times require adjustment of data to a common time point to standardize population biomarker data, in order to reduce measurement error and enable comparisons across measurements. Collection of saliva samples at the same time of day does not resolve this situation if there is variability in the time at which participants first saw daylight. Results of this study suggest that a significant percentage of variation within previous biomarker studies involving SC and sIgA concentrations could be attributed to these biological factors. It is highly recommended that all studies involving stress biomarkers adjust more rigorously for the effect of daily rhythm.

## Ethics Statement

This study has been approved by the Human Research Ethics Committee at the Australian Catholic University. In the event that you have any complaint or concern, or if you have any query that the Investigator has not been able to satisfy, you may write to the Chair of the Human Research Ethics Committee care of the nearest branch of the Research Services Office.

## Author Contributions

BP: the original conception and design of the study, acquisition of data, assay procedure and analysis, drafting the research paper, editing and final approval of the version to be submitted. WS: conception and design of the study, acquisition of data, analysis and interpretation of data, design of the research paper, drafting, editing, and revising the paper critically for important intellectual content and final approval of the version to be submitted. RL: conception of the study, assay procedure and analysis, drafting the research paper, editing and final approval of the version to be submitted. PP: conception of the research paper, statistical analysis and interpretation of results, editing and revising the paper critically for important intellectual content and final approval of the version to be submitted. G-JP: conception and re-design of the research paper, interpretation of data, editing and revising the paper critically for important intellectual content and final approval of the version to be submitted.

## Conflict of Interest Statement

The authors declare that the research was conducted in the absence of any commercial or financial relationships that could be construed as a potential conflict of interest.
